# Substrate flexibility of *Mycoplasma fermentans* mf1 phosphorylcholine transferase

**DOI:** 10.1007/s10719-025-10181-2

**Published:** 2025-03-22

**Authors:** Lena Nuschy, Biswajit Sarkar, Alla Zamyatina, Iain B. H. Wilson

**Affiliations:** 1 Institute of Biochemistry, University of Natural Resources and Life Sciences, Muthgasse 18, Vienna, 1190 Austria; 2 Institute of Organic Chemistry, University of Natural Resources and Life Sciences, Muthgasse 18, Vienna, 1190 Austria

**Keywords:** Phosphorylcholine, Phosphocholine, PC-transferase, *Mycoplasma fermentans*, LicD

## Abstract

Zwitterionic modifications of glycans such as phosphorylcholine or phosphoethanolamine occur in a wide range of prokaryotic and eukaryotic organisms and are known for interaction with the mammalian immune system. Unlike the biosynthesis of membrane phospholipids which is well elucidated, very little is known about the transfer of zwitterionic phosphodiester moieties onto glycoconjugates. The presence and function of relevant enzymes has been suggested by gene knockout or mutation and corresponding aberrant phosphorylcholine metabolism. In the current study, the *Mycoplasma fermentans* phosphorylcholine transferase mf1, with previously confirmed in-vitro activity synthesizing phosphorylcholine-α-glucosyl-1,2-dipalmitoyl glycerol, is demonstrated to not only transfer phosphorylcholine but also phosphoethanolamine from CDP-ethanolamine. Moreover, mf1 is capable of using the β-configuration of the presumed natural substrate but transfers neither to simpler substrates with glucose moieties such as β-D-octyl-glucopyranoside nor to an extended lipid substrate with an additional galactose residue. These findings suggest a certain, but limited, substrate flexibility for bacterial PC-transferases. Mf1 activity is inhibited by β-glycerophosphate, an isomer of part of CDP-glycerol which is known to compete with CDP-ribitol in enzymatic reactions catalyzed by fukutin, a human protein sharing structural homology with mf1. For the first time, a phosphorylcholine transferase, mf1, could be biochemically characterized in vitro and its lipid products with zwitterionic phosphodiesters attached could be detected specifically with the pentraxin serum amyloid P.

**Supplementary Information:**

The online version contains supplementary material available at 10.1007/s10719-025-10181-2.

## Introduction

Phosphorylcholine (PC) and -ethanolamine (PE) are probably best-known as zwitterionic head groups of the phospholipids phosphatidylcholine and -ethanolamine. Both are highly abundant in the eukaryotic cell membrane while only 10% of bacteria are estimated to have phosphatidylcholine incorporated in their membranes [1]. Other molecules harboring PC and PE are platelet-activating factor (1-*O*-alkyl-2-acetyl-*sn*-glycero-3-phosphorylcholine), with a crucial role in human inflammation [2] and certain lipopolysaccharides, where phosphorylcholine controls pathogen virulence [3]. In plant sap, phosphorylcholine is found in a free form [4]. Also a wide range of invertebrates display either PC or PE on protein- or lipid-linked glycans [5].

While pathways of biosynthesis of phosphatidylcholine and phosphatidylethanolamine in eukaryotes [6] and in bacteria [1] are well established, the transfer of PC and PE to glycoconjugates is less elucidated. Four pathways of phosphorylcholine biosynthesis are described, which were investigated by Zhang and colleagues in silico regarding the mechanism of subsequent incorporation in bacterial surface decorations [7]. Enzymes transferring PC to glycoconjugates have been identified by genomic analyses from the *lic* locus that certain PC-harboring organisms have in common, the licD family, whose function and occurrence throughout different species is also reviewed by Young et al. [8].

So far, unlike studies verifying presence or absence of phosphorylcholine in bacterial structures by knock-out of putative phosphorylcholine transferase genes [9], comprehensive in-vitro characterization of such enzymes is lacking. To our knowledge, the only study in which a recombinant phosphorylcholine transferase was confirmed by in-vitro activity is that on mf1 [10]. In their work, they cloned the *mf1* gene from *Mycoplasma fermentans*, a bacterium lacking a cell wall, which was first detected and isolated from a male urogenital tract [11]. The bacterium has been associated with various disorders including its controversial role in the development of AIDS [12]. Moreover, essential lipid membrane components of the bacterium, namely glycoglycerophospholipids (GGPLs), which are however unique to *M. fermentans* [13], were found in synovial fluids of rheumatoid arthritis patients, suggesting it to be a causal agent of RA although the exact mechanism remains to be elucidated [14]. Originally, mf1 was concluded to use as substrate an intermediate of the glycoglycerophospholipid (GGPL) biosynthesis, α-glucosyldiacyl glycerol formed by an enzyme called mf3 [15]. Here, we re-investigate the activity of mf1 and its mode of action performing a variety of biochemical tests. The overall goal is to better understand this abundant, but poorly characterized, group of enzymes in a broad range of organisms in the future.

## Materials and methods

### Cloning of mf1

Gene sequence (GenBank: AB457170.1), codon-optimized for *E. coli* and inserted in pUC57-mini vector, was purchased from Genscript (Genscript Biotech Corp., Piscataway Township, NJ, USA). For cloning into pET151 (Thermo Fisher Scientific, Waltham, MA, USA) with the NEBuilder^®^ HiFi DNA Assembly Cloning Kit (New England Biolabs, Beverly, MA, USA), the *mf1* gene and pET151 were amplified by PCR using the respective overlapping primers Mf1f.

(5’-CTGTATTTTCAGGGAATTGATCCCTTCACCATAAAGACAATGAATGAAAA-3’),

Mf1r (5’- CCGGATCTGAGCTCGCCCTTTTACTTGTCGTTCTTCTTCG-3’),

pET151f (5’- AAGGGCGAGCTCAGATCCG-3’) and pET151r.

(5’- GGTGAAGGGATCAATTCCCTGAAAATACA-3’) and the Q5^®^ High-Fidelity DNA Polymerase (New England Biolabs). PCR conditions were 98 °C for 30 s followed by 35 cycles of 98 °C for 20 s, 70 °C for *mf1* and 63 °C for pET151 respectively for 30 s and 72 °C for 4 min and final extension of 72 °C for 7 min. 50 ng pET151 and 2:1 *mf1* DNA mass were applied in the cloning reaction and NEB^®^ 5-alpha competent *E. coli* transformed with the product. A positive clone was cultured in Luria-Bertani (LB) medium containing 50 µg/mL ampicillin at 37 °C for 16 h and the plasmid isolated using the PureYield™ Plasmid Miniprep System (Promega Corp. Fitchburg, WI, USA). Correct insertion of the *mf1* sequence in frame with the His-Tag was verified by DNA sequencing.

### Expression of mf1

One Shot™ BL21-AI™ Chemically Competent *E. coli* (Thermo Fisher Scientific) were transformed with pET151-mf1 and cultured in 10 mL LB medium supplemented with 50 µg/mL carbenicillin at 37 °C until OD_600_ of 0.6 before the temperature was reduced to 30 °C and expression induced with 0.5 mM IPTG and 0.2% L-arabinose and continued for 5 h. Cells were harvested by centrifugation at 6000 g for 15 min, resuspended in 50 mM Tris-HCl buffer pH 8, and lysed using a vibration mill (MM400, Retsch GmbH, Haan, Germany) with 100 mg ϕ0.1 mm glass beads (30/s for 3 min twice) combined with 3 freeze-thaw cycles in liquid nitrogen and 42 °C respectively. The homogenate was centrifuged at 15,000 g for 5 min and protease-inhibitor added to the supernatant (termed as lysate mf1). After separation on 10% SDS-polyacrylamide gel and transfer to a nitrocellulose membrane (Pall Corporation, Port Washington, NY, USA), mf1 was detected in the cell lysate by Western Blot using an anti-His antibody (clone HIS-1, 1:10000 dilution. Sigma-Aldrich, Darmstadt, Germany).

### Lipid substrate synthesis

Synthesis of glucopyranosyl glycerolipid is reported in the literature. Khan and co-workers reported the selective synthesis of the α-anomer of the glycerolipid using silyl protecting group to perform glycosylation with solketal [16]. Further acylation reaction followed by hydrogenolysis reaction afforded the desired glycolipid. Isaad and colleagues synthesized the β-anomer of the glycolipid by changing the protecting group to–OAc [17].

As for our study both the α- and β-anomer were desirable, we used the benzyl protecting group which provided both anomers, but with preferential α-anomeric selectivity. The glycosyl donor (2,3,4,6-tetra-O-benzyl thiotolyl-β-D-glucopyranoside) was synthesized from D-glucopyranose using previously reported procedure. Then the glycosylation of the donor thioglycoside and the glycerolipid was performed at 0 °C in dichloromethane. We obtained both the α- and β-anomer (α:β = 8:5) with 70% yield. The desired deprotected lipids were afforded using hydrogenolysis in presence of Pd Black with a quantitative yield. Synthesized lipids were confirmed by comparing the NMR spectra (Supplementary Information) with previous reports of the same molecules.

### Activity assay

Activity of mf1 was first confirmed by an assay similar to the one described [10] before further biochemical characterization experiments were performed. In detail, 1 mM α- and β-glucosyldipalmitoyl glycerol, respectively were re-dissolved for a small scale in-vitro assay in 100 mM ammonium carbonate buffer pH 8. The assay included further 10 mM 2-mercaptoethanol (β-ME), 7.5 mM MgCl_2_, 2.5 mM CDP-choline and 3 µL of mf1 lysate. The ability of mf1 to transfer also phosphoethanolamine was tested using CDP-ethanolamine instead of CDP-choline as donor substrate. All incubations were carried out at 37 °C for 16 h, if not stated different. Samples were mixed 1:1 with 2,5-dihydroxy-acetophenone matrix (2,5-DHAP), spotted on a MTP 384 target plate ground steel BC (Bruker Daltonics Corporation, Billerica, MA, USA) and analyzed by MALDI-TOF MS and MS/MS using a rapifleX^®^ TOF/TOF (Bruker).

### Removal of PC and extension of acceptor substrate

After efficient PC transfer was confirmed, homemade recombinant phosphodiesterase (unpublished data), acting in a similar manner to an enzyme described [18], was added to the reaction mixture and incubated again. Samples were analyzed by MALDI-TOF MS as before. In order to extend the acceptor substrate by another monosaccharide, β-1,4-galactosyltransferase (GalT from bovine milk, Sigma-Aldrich, Darmstadt, Germany) was added to the re-dissolved lipid in an assay with 0.8 mM UDP-Gal and 8 mM MnCl_2_. After incubation, the extension of the sugar moiety by one galactose was confirmed by mass spectrometry, before lysate mf1, MgCl_2_, β-ME and CDP-choline were added as described and the incubation continued. Furthermore, a GalT assay was subsequently performed after completed PC-transferase activity assay and samples measured after incubation. Finally, flexibility of acceptor substrate was further tested with β-D-octyl-glucopyranoside.

### Biochemical characterization of mf1

Assays to determine the dependence of mf1 on pH, temperature, buffer salts or cations were performed in triplicates and contained the respective varying parameter while all other conditions were kept constant. First, the buffer was changed from ammonium carbonate to Tris/HCl, AMPD, McIlvaine or HEPES, then a pH range of 6.5–9.5 was covered using AMPD (2-amino-2-methyl-1,3-propanediol) buffer. The divalent metal ion was changed to Ca^2+^, Mn^2+^, Ni^2+^ or Zn^2+^. Furthermore, activity in presence of EDTA and the potential competitor, β-glycerophosphate (disodium salt hydrate, Sigma-Aldrich, Darmstadt, Germany; 5 mM) was tested. To evaluate enzymatic activity of mf1 at different temperatures, assays were incubated at 4 °C, 18 °C, 25 °C, 30 °C, 37 °C, 50 °C and 70 °C. Finally, product formation should be followed over time, therefore samples were measured after 0.5 h, 1 h, 2 h, 4 h. Results are shown as ratio of product to remaining substrate, calculated from the signal intensity values at *m/z* 896 and 753 respectively (processed with Bruker Flexanalysis 3.3.80 software).

### Dot blots

1 µL of assays +/- mf1 and subsequent incubation was spotted on nitrocellulose membrane (Pall). After complete absorption, membranes were blocked for 1 h in TBS-T buffer + 0.5% BSA (Carl Roth Corporation, Karlsruhe, Germany) and further processed as described for Western Blots. Blots were challenged with serum amyloid protein (Fitzgerald, Acton, MA; 1:200), C-reactive protein (MP Biochemicals, Santa Ana; 1:200, supplemented with 2.5 mM CaCl_2_) and TEPC-15 (IgA-κ from murine myeloma, clone TEPC-15, Sigma-Aldrich, Darmstadt, Germany; 1:200), followed by the relevant antibodies and developed with SigmaFAST BCIP/NBT [19].

### Structural modeling

Common databases and programs including Protein Data Bank [20], Phyre2 [21], AlphaFold 3 [22, 23] and SWISS-MODEL [24] were searched for available structures and structural homologs of mf1 using the amino acid sequence as query. Structures were visualized and analyzed by PyMOL by Schrödinger [25]. A BLASTP search using the amino acid sequence of mf1 was conducted and five proteins from different *Mycoplasma* species and other bacteria selected for further comparison, sharing between 53% and 28% sequence identity (WP_349222499, WP_063626040, WP_027334422, MFR1766058, MBP3452833). Sequence alignments also including FKRP (PdB: 6KAJ) were performed using the MEGA11 software [26] with the ClustalW and the MUSCLE algorithm and default settings.

## Results

### Expression of mf1

**Fig. 1:**
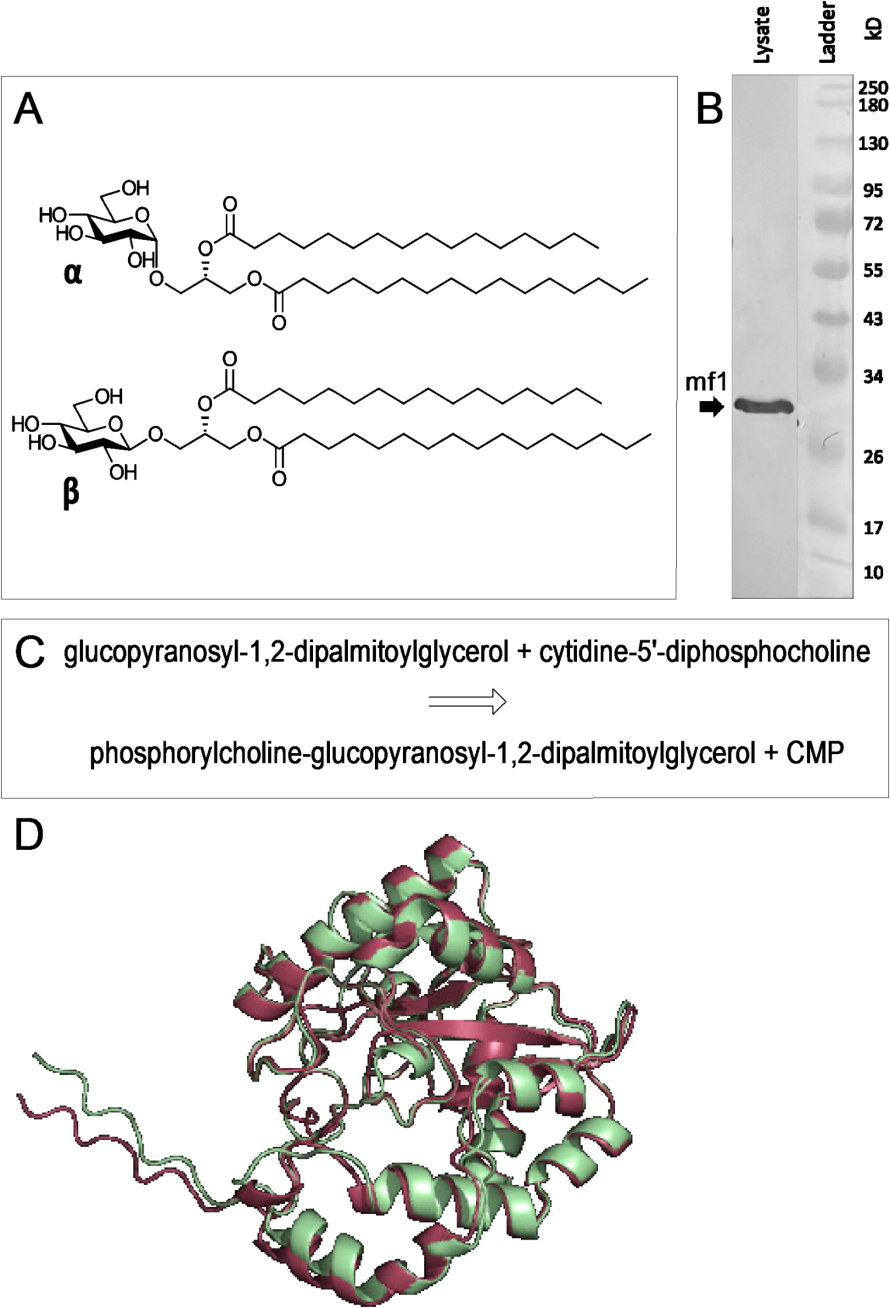
**A**: Chemical structure of both lipid substrates synthesized for enzymatic activity and characterization experiments (upper structure: α-anomer, lower structure: β-anomer). **B**: Western Blot of the BL21-AI *E. coli* cell lysate using an anti-His antibody for detection. A distinct band at approximately 31 kD is indicated with an arrow. **C**: Putative reaction catalyzed by mf1 resulting in phosphorylcholine-glucopyranosyl-1,2-dipalmitoylglycerol and cytidine monophosphate (CMP). **D**: Structural model of mf1 predicted by AlphaFold 3. Protein sequence search suggested two models, cholinephosphotransferase mf1 (red) and an uncharacterized protein of Mycoplasma fermentans PG18 (green). Both models were superimposed and depicted using Schrödinger PyMOL (RMSD 0.454). C-terminus is shown on the left, region of beta sheets surrounded by alpha helices on the right

The synthesized DNA sequence encoding mf1 was firstly cloned into pET151 expression vector, before transformation of BL21-AI *E. coli* with the plasmid. After expression for 5 h at 30 °C and subsequent cell lysis, mf1 could be detected in the cell lysate via the N-terminal His-tag by Western Blot using an anti-His antibody (Fig. 1b) as a clear band at approximately 31 kDa.

According to searches of models and actual crystallographic structures, mf1 shares closest structural homology with human fukutin-related protein, a ribitol transferase from the LicD family, a tetrameric enzyme defective in one form of muscular dystrophy [27]. According to a Phyre2 Structural Homology search, even though only 58% of the protein sequence could be covered, the two proteins share only 21% sequence identity. Beyond that, no confident structural homologs were found, suggesting that mf1 belongs to a novel structural group of PC-transferases of the LicD family, as yet largely uncharacterized. To date, the protein structure of mf1 was not experimentally determined; however an AlphaFold 3 search revealed two relevant computed structures, cholinephosphotransferase mf1 and an uncharacterized protein of *Mycoplasma fermentans* PG18, from which the gene sequence of Mf1 was originally extracted. Both models share the same amino acid sequence and are predicted structures of mf1 (RMSD of superimposition was 0.454) with a confined region of beta-sheets surrounded by mainly alpha-helices and minor deviations in the C-terminus (Fig. 1d, overlay of AlphaFold 3 models). A brief sequence alignment with selected proteins from a BLASTP search to mf1 revealed a highly conserved region between Tyr16 and Tyr69 forming a structure of alternating α-helices and β-sheets (α-β-α-β-α). Further, this motif includes four aspartic acid residues, Asp50, Asp51, Asp52 and Asp54, found in all selected sequences (Supplementary Information), potentially indicating a role in enzymatic activity; whereas Asp52, and Asp50 depending on the processing algorithm, even align with fukutin-related protein (FKRP). The hydrophobic hG[GS] motif, which was found to be highly conserved in FKRP from different organisms [27] is absent from mf1 related proteins of the licD family.

### PC-Transferase activity and substrate variation (transfer of zwitterionic moieties)

**Fig. 2:**
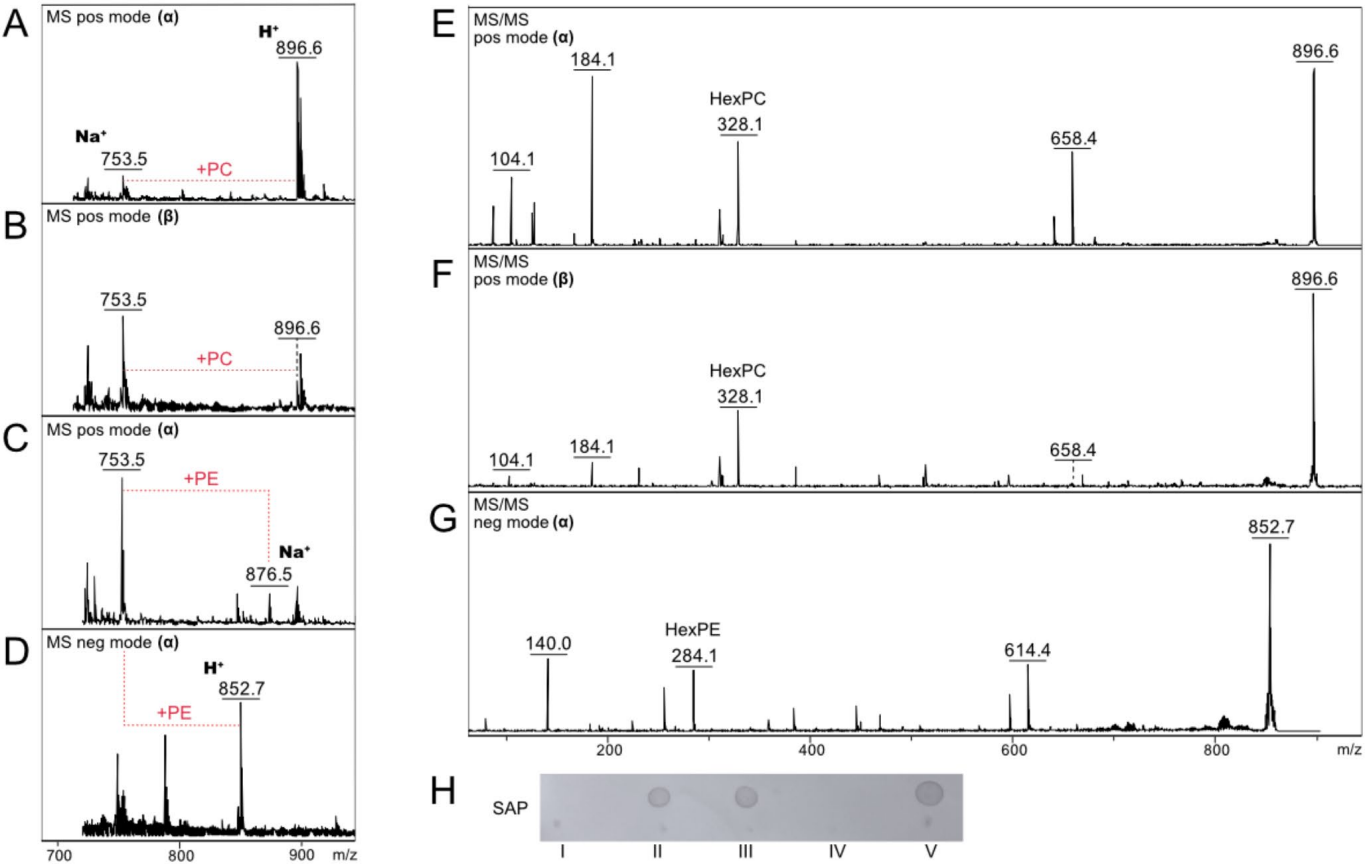
Transferase activity of mf1 in *E. coli* BL21-AI cell lysate. Assays were incubated for 16 h at 37 °C and samples measured by MALDI-TOF MS and MSMS. **A** and **B**: Phosphorylcholine transferase activity assay using α- or β-glucosyldipalmitoyl glycerol as substrate, respectively (*m/z* 753 corresponds to the lipid substrate, sodium adduct; successful transfer of phosphorylcholine indicated as *m/z* 896; H^+^). **C** and **D**: Phosphoethanolamine transferase activity assay using α-glucosyldipalmitoyl glycerol as substrate. Samples were measured in positive and negative ion mode MS (successful transfer indicated as *m/z* 876; sodium adduct, in positive and *m/z* 852 in negative MS corresponding to phosphoethanolamine-α-glucosyldipalmitoyl glycerol). **E** and **F**: MSMS of *m/z* 896 phosphorylcholine-α- and β-glucosyldipalmitoyl glycerol, respectively. Inverse ratios of signal intensities of fragment ions at *m/z* 184 (PC-related, PC + H_2_O) and 328 (HexPC) depending on the configuration of the substrate were observed. G: Negative ion mode MSMS of *m/z* 852, phosphoethanolamine-α-glucosyldipalmitoyl glycerol, whereby the fragment ions at *m/z* 140 & 284 are PE + H_2_O or HexPE. **H**: Serum amyloid protein (SAP) dot blot of mf1 assays. Assays were performed and incubated as described, before 1 µL was loaded on nitrocellulose membrane. I and IV: α- and β-glucosyldipalmitoyl glycerol containing assay, respectively, without enzymatic treatment. II: α-configuration of substrate + CDP-choline after enzymatic treatment with mf1. III: α-configuration of substrate + CDP-ethanolamine after enzymatic treatment with mf1. V: β-configuration of substrate + CDP-choline after enzymatic treatment with mf1

The ability of mf1 in the cell lysate to transfer phosphorylcholine from donor substrate CDP-choline to the acceptor lipid glucosyldipalmitoyl glycerol (Fig. 1c predicted reaction) was confirmed by mass spectrometry. An α- and β-configuration of the lipid substrate (chemical structures Fig. 1a, m*/z* 753, sodium adduct; Fig. 2a and b), both chemically synthesized, were tested. Product formation after 16 h of incubation at 37 °C was visible as *m/z* 896 [10]. For both substrates, MS/MS spectra were as expected (fragment ions at *m/z* 104, 184, 328 and 658 corresponding to choline, phosphorylcholine, hexose-phosphorylcholine and the lysolipid, respectively). The ratio of signal intensities of fragment ions varied depending on the linkage of the glucose residue, phosphorylcholine-β-glucopyranosyldipalmitoyl glycerol, has a strong fragment ion at *m/z* 328, while the ratio is on the side of *m/z* 184 for the α-configuration (Fig. 2e and f).

Besides the phosphorylcholine transferase activity of mf1, the ability to transfer phosphoethanolamine from a similar donor, CDP-ethanolamine, onto the glucose moiety was tested. Indeed, in negative ion mode, phosphoethanolamine-glucosyldipalmitoyl glycerol (*m/z* 852; *m/z* 876 sodium adduct in positive ion mode; fragment ions in negative mode 140, 284 and 614 corresponding to phosphoethanolamine, hexose-phosphoethanolamine and the lysolipid, respectively) could be detected, however only when the α-configuration of the substrate was used (Fig. 2c, d and g). When CDP-choline and CDP-ethanolamine were added to the assays simultaneously, mf1 was able to synthesize both types of product, however it cannot be estimated which donor substrate or reaction is preferred, since the PE-modification is only well visible in negative ion mode, while the PC-product appears exclusively in positive ion mode.

Since CDP-glycerol (CDP-Gro) is a known inhibitor in synthesis pathways catalyzed by the structural homologs of mf1, i.e. fukutin and fukutin-related protein [28], PC-transferase activity of mf1 in the presence of 5 mM β-glycerophosphate (i.e., glycerol-2-phosphate, an isomer of part of CDP-Gro) was investigated. Indeed, the effect was similar as product formation was reduced by 70% (data not shown).

In addition to MALDI TOF MS, product formation and recognition by different proteins with known ability to bind zwitterionic moieties was investigated by dot blots. Glucosyldipalmitoyl glycerol with and without PC and PE modification adsorbed to nitrocellulose membrane was challenged. PC-products of both substrate configurations as well as phosphoethanolamine-α-glucosyldipalmitoyl glycerol were selectively recognized by Serum amyloid protein (SAP) (Fig. 2h). Binding of the PC-recognizing antibody TEPC-15 was very weak, C-reactive protein (CRP) showed unspecific binding to both, substrate and product (data not shown).

**Fig. 3:**
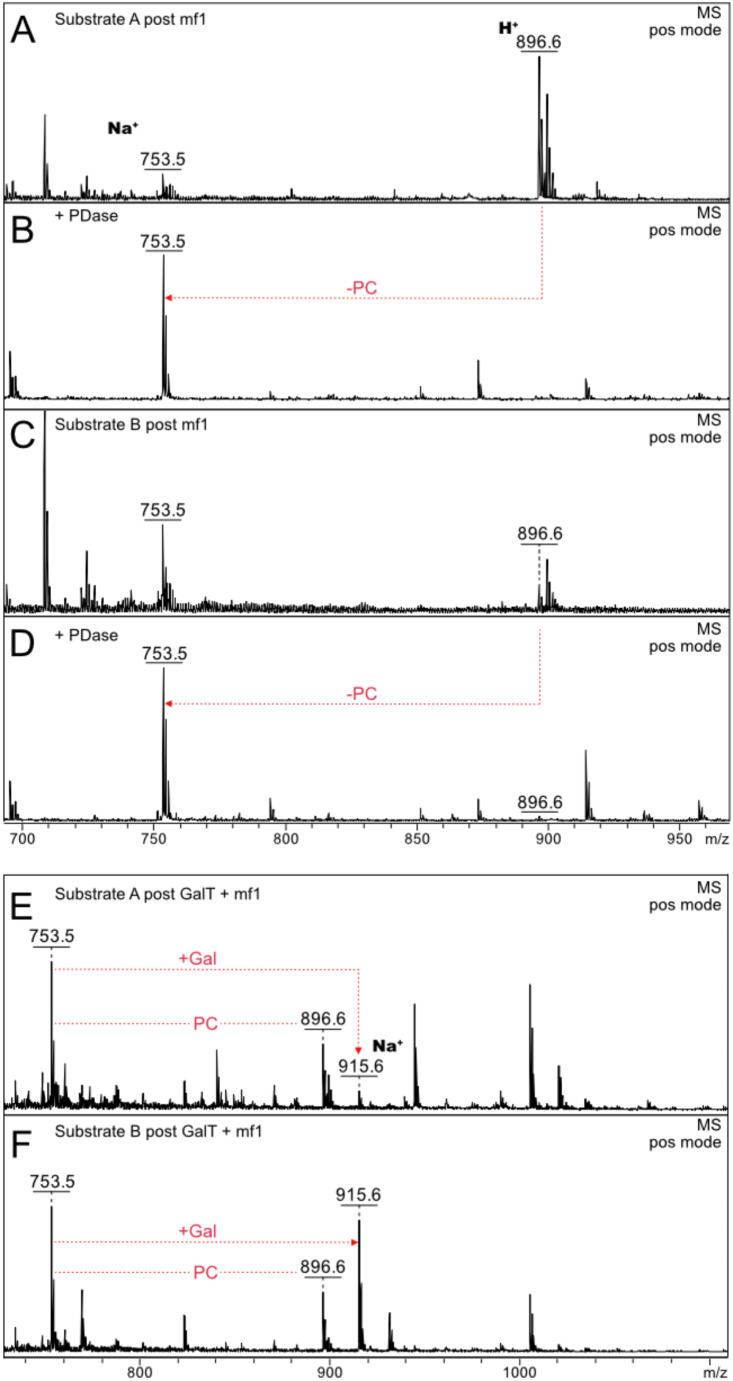
Removal of phosphorylcholine by in-house expressed PDase with similar reaction mechanism to an enzyme described [18]. Assays were first incubated with mf1, phosphorylcholine-product formation confirmed and afterwards treated with PDase, incubated at 37 °C for 16 h and samples analyzed by MALDI-TOF MS. **A**-**D**: Spectra of α-glucosyldipalmitoyl glycerol and β-glucosyldipalmitoyl glycerol post mf1 phosphorylcholine transfer before and after PDase addition, respectively. Efficient removal of phosphorylcholine from the glucose moiety is visible as decline or absence of the signal at *m/z* 896 after PDase addition. **E** and **F**: Activity assay of mf1 on the glucosyl-substrates extended by one galactose residue. α- and β-glucosyldipalmitoyl glycerol substrates were treated with β-1,4-galactosyltransferase from bovine milk for 16 h at 37 °C before mf1 was added to the reaction. Successful transfer of galactose visible as *m/z* 915 (sodium adduct); activity of mf1 detected exclusively on the remaining substrate without galactose (product at *m/z* 896 as in Fig. 2). Inverse ratios of product formation, galacto-glucosyldipalmitoyl glycerol and phosphorylcholine-glucosyldipalmitoyl glycerol for α- and β- substrate configurations are observed

### Removal of PC and extension of acceptor substrate

Confirmation of the transfer of PC was shown by the addition of a phosphodiesterase (expressed in-house) to the mf1 assay after completed reaction. The product ion at m/z 896 almost disappeared after incubation for 16 h while the substrate ion at m/z 753 increased in intensity (Fig. 3a-d).

Mf1 activity on extended acceptor substrate was tested by addition of one galactose to glucose by a β-1,4-galactosyltransferase in a first reaction (*m/z* 915, sodium adduct) that was continued by the mf1 assay afterwards (Fig. 3e and f). Although mf1 was still able to partially convert the remaining substrate without galactose to phosphorylcholine-glucosyldipalmitoyl glycerol, no PC-transfer to the lipid with the attached disaccharide could be observed. More galactose-glucosyldipalmitoyl glycerol compared to phosphorylcholine-glucosyldipalmitoyl glycerol was generated for the substrate in β-configuration while the ratio was inverse when the α-configuration was applied indicating higher efficiency of mf1 on the α-substrate compared to preference of the β-substrate by the galactosyltransferase.

Similar results were obtained when the GalT reaction was carried out using the glucosyldipalmitoyl glycerol/phosphorylcholine-glucosyldipalmitoyl glycerol mixture as substrates. Modification of glucose with PC appears to block further addition of galactose, as GalT was active on the glucose substrate without PC but not after attachment of PC.

No phosphorylcholine-transfer was detectable on β-D-octyl-glucopyranoside (data not shown), which suggests that the fatty acid extensions or the glycerol moiety are required for mf1 activity and proper substrate binding.

### Biochemical characterization of mf1

**Fig. 4:**
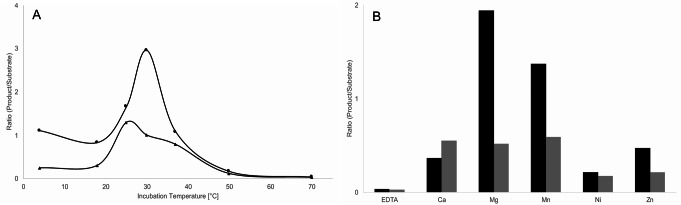
Effects of temperature and different metal ions on mf1 activity. Assays using both, the α- (circles in **A**, black color in **B**) and the β-configuration (triangles in **A**, grey color in **B**) of the substrate were performed as described and incubated for 16 h. Samples were measured by MALDI-TOF MS

Incubation of the enzymatic reaction at different temperatures revealed substantial activity of mf1 at cool environments; at 4 °C the intensities of α-glucosyldipalmitoyl glycerol substrate and phosphorylcholine modified product were still equal (Fig. 4a). Highest amounts of product were formed at 25 °C to 30 °C, in general however, ratios were lower for the β-configuration. On the other hand, mf1 was found heat-sensitive, at 50 °C product formation was diminished, while at 70 °C no phosphorylcholine product could be detected at all.

Dependence of mf1 activity on a divalent cation was confirmed, as product formation in the presence of EDTA was negligible (Fig. 4b). Strong differences in enzymatic activity between substrate configurations were found. Mg^2+^ and Mn^2+^ addition increased product formation by two times higher (α-anomer), while amounts of product were little, when Zn^2+^ or Ni^2+^ were added to the assay and especially the latter appears to inhibit mf1 activity.

**Fig. 5:**
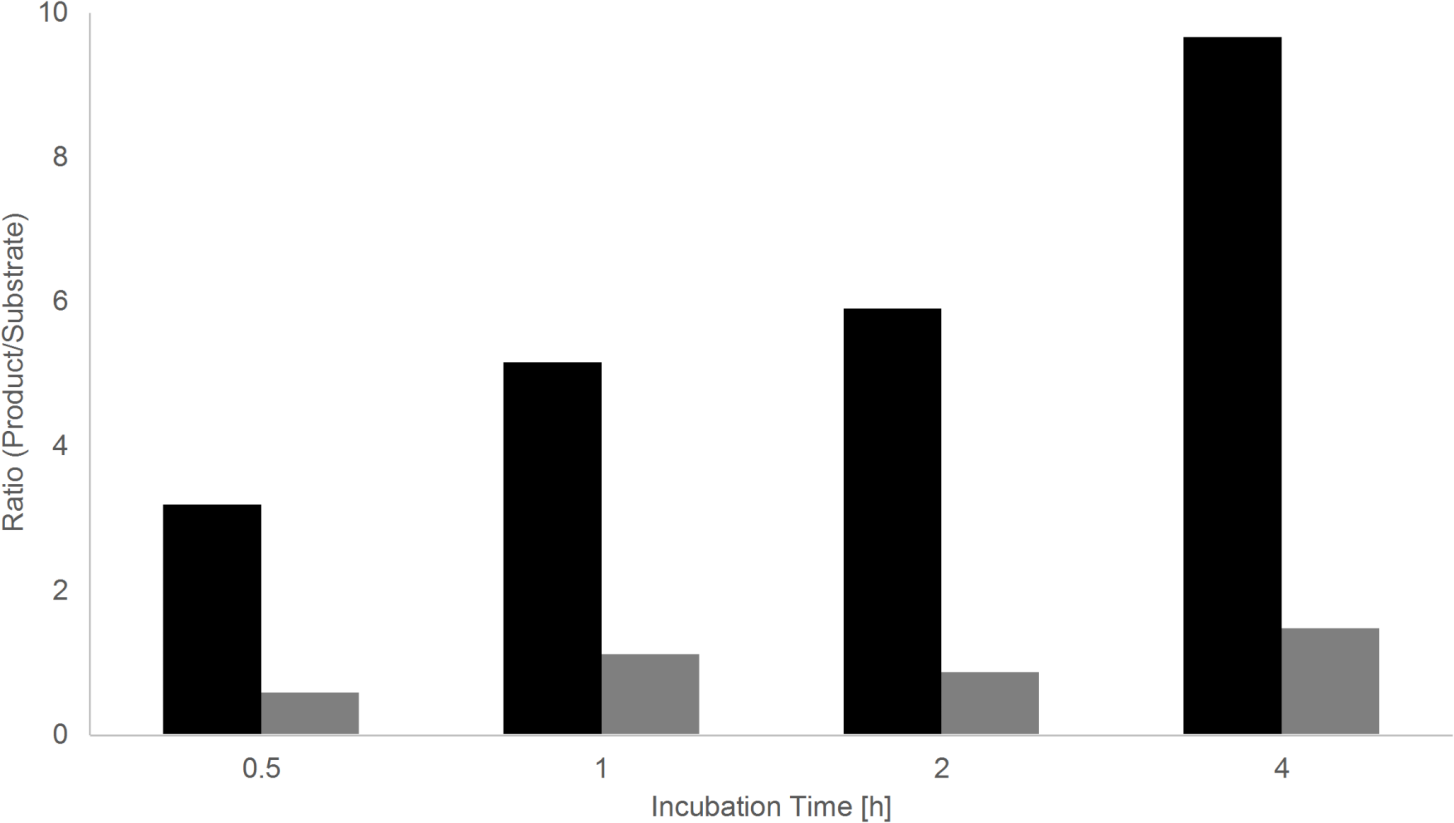
Phosphorylcholine transferase activity of mf1 followed over time. Samples were analyzed by MALDI-TOF MS at different time points and the ratio of product to substrate calculated. Assays were incubated at 37 °C using both, the α- (black) and β-configuration (grey) of the substrate

Following product formation over time revealed efficient conversion in a relatively short time span (Fig. 5); after 30 min, the ratio was more than three times on the side of the phosphorylcholine-α-glucosyldipalmitoyl glycerol and reached the maximum after four hours. At this timepoint, roughly 95% of the original substrate has been converted. Similar trends were observed for both configurations of acceptor substrate, however at different levels.

**Fig. 6:**
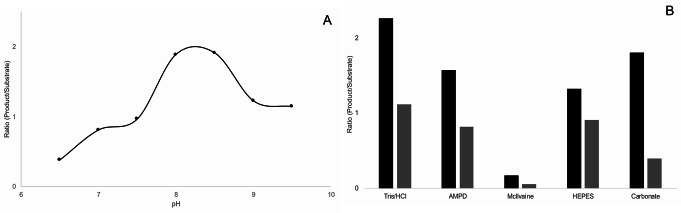
Effects of pH and buffer salts on mf1 activity. **A**: Assays using only the α-configuration of the substrate were performed in AMPD buffer and incubated for 16 h. Samples were measured by MALDI-TOF MS. **B**: Both, the α- (black) and the β-configuration (gray) of the substrate were used

The effect of different buffer salts (all pH 8) on activity of mf1 was investigated (Fig. 6b). Highest levels of substrate conversion (for both configurations of the accceptor substrate, α depicted in black and β in grey) could be achieved with Tris/HCl buffer while the smallest differences between the substrates were found when HEPES buffer was used. Least amounts of product were measured using McIlvaine buffer in the assay, possibly as phosphate is a component of this buffer. AMPD with fair buffer capacity in a rather alkaline pH range was chosen for subsequent determination of pH optima. Therefore, pH was varied from 6.5 to 9.5 in 0.5 increments. For the β-substrate configuration no conclusions concerning the effect of pH on enzymatic activity could be drawn (data not shown); however, using α-glucosyldipalmitoyl glycerol, a clear pH optimum of around 8–8.5 was found (Fig. 6a).

## Conclusions

In summary, we have verified and extended the previous study on the PC-transferase mf1, concluding that the enzyme is able to use different zwitterionic donor substrates. In terms of acceptors, it shows a certain but limited flexibility, where it may prefer the α over β-configuration, while extension of the glucosyldipalmitoyl glycerol with galactose prevents transfer. The enzyme requires a divalent metal ion, since no product could be formed in the presence of EDTA, optimally magnesium or manganese ions. It tolerates a broad range of common buffers except for phosphate buffer, preferring a slightly alkaline pH. Mf1 is sensitive to heat and almost inactive at 50 °C, but on the other hand maintains activity at low temperatures. Sequence alignments with related licD superfamily proteins revealed a highly conserved region rather N-terminal, including four Asp residues, forming an alternating α-β-α-β-α motif, likely to be involved in catalytic activity and cation coordination. We also found that β-glycerophosphate, an isomer of part of CDP-Gro inhibits the PC-transfer by mf1, similar to fukutin and fukutin-related protein. From these results, together with structural homology models, it can be assumed that other members of the licD family might share similar characteristics which will require further clarification in the future.

## Electronic supplementary material

Below is the link to the electronic supplementary material.

Supplementary Material 1

Supplementary Material 2

Supplementary Material 3

## Data Availability

Underlying data is available on request.
