# Supplementary figures and images for: Substrate flexibility of Mycoplasma fermentans mf1 phosphorylcholine transferase
Source: Glycoconj J. 2025 Mar 22;42(2):87–96. doi: 10.1007/s10719-025-10181-2 (PMC11982090; doi:10.1007/s10719-025-10181-2)

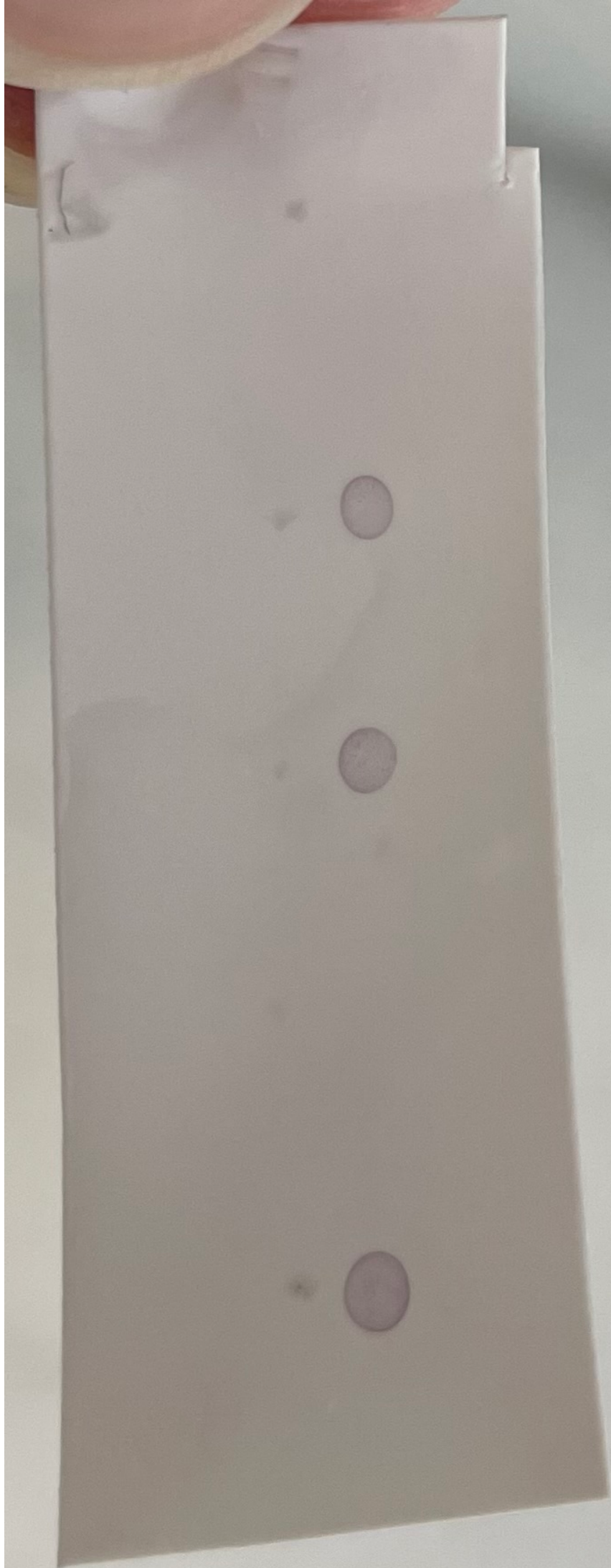

Supplement: Supplementary file 1 — Supplementary Material 1 [file 10719_2025_10181_MOESM1_ESM.pdf]

pos control mf1  
↓

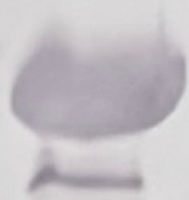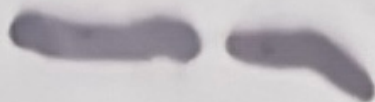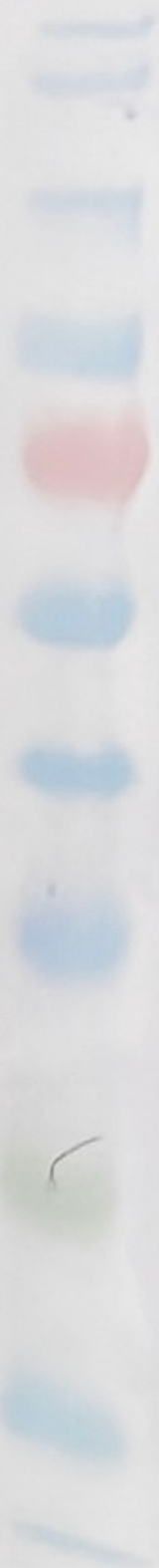

Supplement: Supplementary file 2 — Supplementary Material 2 [file 10719_2025_10181_MOESM2_ESM.pdf]
