# Supplementary material for: Substrate flexibility of Mycoplasma fermentans mf1 phosphorylcholine transferase
Source: Glycoconj J. 2025 Mar 22;42(2):87–96. doi: 10.1007/s10719-025-10181-2 (PMC11982090; doi:10.1007/s10719-025-10181-2)
Supplement: Supplementary file 3 — Supplementary Material 3 [file 10719_2025_10181_MOESM3_ESM.pdf]

Substrate flexibility of *Mycoplasma fermentans* mf1 phosphorylcholine transferase

Lena Nuschy[1], Biswajit Sarkar[2], Alla Zamyatina[2], Iain B.H. Wilson[1]

[1] Institute of Biochemistry, [2] Institute of Organic Chemistry, University of Natural Resources and Life Sciences, Muthgasse 18, 1190 Vienna, Austria

Running title: Phosphorylcholine transferase activity of mf1

Corresponding author: Lena Nuschy, lena.nuschy@boku.ac.at

Supplementary Information

Lipid Substrate Synthesis

The synthesis of both anomers of glucosyl diacylglyceride (Glc-DAG) was performed using a glucose-derived thioglycoside as the donor and di-hexadecanoyl-*sn*-glycerol as the acceptor. A tetrabenzylated thioglycoside **S1** was reacted with commercially available diacylglycerol **S2** using NIS/TfOH as a promoter resulting in the formation of a corresponding glycoside as an anomeric mixture (α/β = 8:5) which were successfully separated by HPLC to give the protected α-glucosyl-diacylglycerol **S3** and β-glucosyl-diacylglycerol **S4** with 43% and 27% yields, respectively (Scheme S1). The anomeric configuration was confirmed using NMR by comparing the heteronuclear C-H coupling constants J_{α} = 168.3 Hz for **S3** and J_{β} = 160.2 Hz for **S4**. Global deprotection by hydrogenolysis on Pd-black gave α-Glc-DAG **1** and β-Glc-DAG **2** with 95% and 91% yield, respectively.

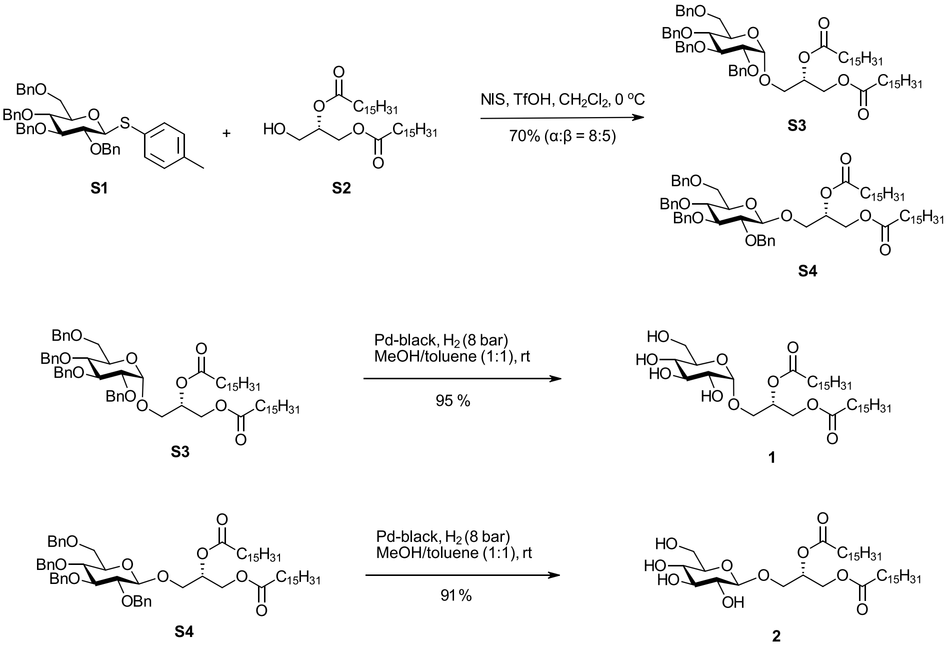

Scheme S1. Synthesis of α-Glc-DAG **1** and β-Glc-DAG **2**

Experimental procedures

General Information

Reagents and solvents were purchased from commercial suppliers and used without further purification unless otherwise stated. Dichloromethane was distilled from CaH_2 and stored over activated molecular sieves (4Å). Other solvents were dried by storage over activated molecular sieves for at least 48 h prior to use. Residual moisture was determined by coloumbometric titration on a Mitsubishi CA21 Karl Fischer apparatus and did not exceed 20 ppm. Reactions were monitored by TLC performed on silica gel 60 F254 HPTLC precoated glass plates with a 25 mm concentration zone (Merck). Spots were visualized by dipping into a sulfuric acid − *p*-anisaldehyde solution and subsequent charring at 250 °C. Solvents were removed under reduced pressure at ≤40 °C. Preparative HPLC was performed on a YMC Pack SIL-06 250 × 20 mm, S-5 µm, 6 nm column or on a YMC Pack SIL-06 250 × 10 mm, S-5 µm, 6 nm column. NMR spectra were recorded at 298 K on a Bruker Avance III 600 spectrometer (^{1}H at 600.22 MHz; ^{13}C at 150.92 MHz) using standard Bruker NMR software. Chemical shifts are reported in ppm. ^{1}H-NMR, ^{13}C-NMR are referenced to residual solvent signals (for MeOD: 3.31 ppm, for $CDCl_3$: 7.26 ppm).

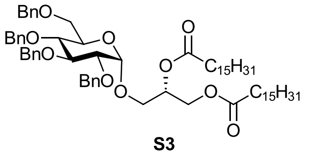

S3

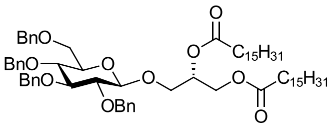

S4

3-*O*-(2,3,4,6-tetra-*O*-benzyl-α-D-glucopyranosyl)-1,2-di-*O*-hexadecanoyl-*sn*-glycerol (S3) and **3-*O*-(2,3,4,6-tetra-*O*-benzyl-β-D-glucopyranosyl)-1,2-di-*O*-hexadecanoyl-*sn*-glycerol (S4)**

A solution of **S1**[1,2] (20.5 mg, 0.0317 mmol) and **S2** (9 mg, 0.0158 mmol) in CH_2Cl_2 (5 ml) was stirred with molecular sieves (3Å, 30 mg) for 15 min under atmosphere of Ar. *N*-Iodosuccinimide (8 mg, 0.035 mmol) and trifluoromethanesulfonic acid (0.21 µl, 0.0032 mmol) were added successively at 0 °C, and the stirring was continued for 2 h. The reaction mixture was brought up to r.t. and quenched by addition of satd. aq. $NaHCO_3$ (1 ml) and satd. aq. $Na_2S_2O_3$ (1 ml). The reaction mixture was diluted with CH_2Cl_2 (30 ml) and washed successively with satd. aq. $Na_2S_2O_3$ (2 x 10 ml), satd. aq. $NaHCO_3$ (2 x 10 ml), and brine (2 x 15 ml). The organic layer was dried over Na_2SO_4 and concentrated. The residue was purified by column chromatography on silica gel (HPLC; toluene – EtOAc, 95:5) to give **S3** (7.4 mg, 43%) and **S4** (4.6 mg, 27%). Analytical data were in accordance with the previously published data.[3,4]

S3: ^{1}H NMR (600 MHz, $CDCl_3$): δ [ppm] = 7.34-7.24 (m, 18H), 7.14-7.12 (m, 2H), 5.26-5.22 (m, 1H), 4.95 (d, 1H, *J* = 10.9 Hz), 4.82 (d, 1H, *J* = 10.9 Hz), 4.79 (d, 1H, *J* = 10.9 Hz), 4.75 (s, 1H), 4.74 (d, 1H, *J* = 8.2 Hz), 4.62 (d, 1H, *J* = 12.0 Hz), 4.59 (d, 1H, *J* = 12.1 Hz), 4.47 (d, 1H, *J* = 8.0 Hz), 4.45 (d, 1H, *J* = 9.3 Hz), 4.40 (dd, 1H, *J* = 3.7 Hz, *J*=11.9 Hz), 4.18 (dd, 1H, *J* = 6.1 Hz, *J* = 12 Hz), 3.93 (t, 1H, *J* = 9.2 Hz), 3.76-3.69 (m, 3H), 3.65-3.59 (m, 2H), 3.56-3.53 (m, 2), 2.29-2.25 (m, 4H), 1.59-1.56 (m, 4H), 1.30-1.24 (m, 48H), 0.88 (t, 6H, *J*=6.9 Hz); ^{13}C NMR (151 MHz, $CDCl_3$): δ [ppm] = 173.33, 173.00, 138.83, 138.29, 137.90, 128.45, 128.37, 128.34, 127.94, 127.86, 127.84, 127.82, 127.70, 127.65, 127.56, 97.76, 81.83, 80.09, 77.50, 75.68, 75.04, 73.50, 73.11, 70.58, 69.86, 68.40, 66.42, 62.49, 34.30, 34.13, 31.93, 29.71, 29.67, 29.52, 29.37, 29.32, 29.17, 29.14, 24.92, 24.90, 22.69, 14.12.

S4: ^{1}H NMR (600 MHz, $CDCl_3$): δ [ppm] = 7.35-7.25 (m, 18H), 7.15-7.14 (m, 2H), 5.29-5.26 (m, 1H), 4.92 (d, 1H, *J*=11.0 Hz), 4.91 (d, 1H, *J*=10.9 Hz), 4.80 (d, 1H, *J*=10.8 Hz), 4.77 (d, 1H, *J*=10.9 Hz), 4.69 (d, 1H, *J*=11 Hz), 4.61 (d, 1H, *J*=12.2 Hz), 4.54 (d, 1H, *J*=12.2 Hz), 4.52 (d, 1, *J*=10.8 Hz), 4.41-4.21 (m, 2 H), 4.22 (dd, 1H, *J*=6.7 Hz, *J*=11.9 Hz), 4.06 (dd, 1H, *J*=4.68 Hz, *J*=10.8 Hz), 3.73-3.66 (m, 3H), 3.64-3.58 (m, 2H), 3.45-3.41 (m, 2H), 2.29-2.24 (m, 4H), 1.60-1.56 (m, 4H), 1.30-1.25 (m, 48H), 0.88 (t, 6H, *J*=7.1 Hz); ^{13}C NMR (151 MHz, $CDCl_3$): δ [ppm] = 173.37, 173.02, 138.38, 138.09, 128.38, 128.36, 128.08, 127.96, 127.82, 127.77, 127.66, 127.64, 127.56, 103.87, 84.58, 81.98, 77.69, 75.67, 75.02, 74.96, 74.75, 73.52, 70.00, 68.78, 67.93, 62.69, 34.30, 34.13, 31.93, 29.71, 29.67, 29.52, 29.37, 29.31, 29.16, 29.12, 24.94, 24.90, 22.69, 14.12.

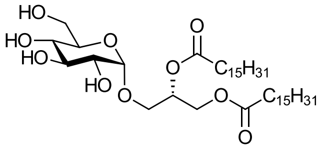

1

3-*O*-α-D-glucopyranosyl-1,2-di-*O*-hexadecanoyl-*sn*-glycerol (1, α-Glc-DAG)

To a solution of **S3** (7.4 mg, 0.007 mmol) in MeOH/toluene (1:1, 5 mL) was hydrogenated over Pd-black (23 mg) at 8 bar for 24 h using high pressure hydrogenation reactor. The solids were removed by filtration over a pad of Celite and the filtrate was concentrated to give **1** (4.7 mg, 95%). Analytical data were in accordance with the previously published data.[3]

^{1}H NMR (600 MHz, $CDCl_3/CD_3OD$, 3:1): δ [ppm] = 5.27-5.26 (m, 1H), 4.81 (d, 1H, *J*=3.9 Hz), 4.48 (dd, 1H, *J*=3.12 Hz, *J*=12.1 Hz), 4.20 (dd, 1H, *J*=6.5 Hz, *J*=12.1 Hz), 3.87 (dd, 1H, *J*=5.6 Hz, *J*=10.7 Hz), 3.80 (dd, 1H, *J*=2.5 Hz, *J*=11.8 Hz), 3.71 (dd, 1H, *J*=5.16 Hz, *J*=11.8 Hz), 3.66-3.62 (m, 2H), 3.59-3.56 (m, 1H), 3.41 (dd, 1H, *J*=3.7 Hz, *J*=9.72 Hz), 2.33 (q, 4H, *J*=7.38 Hz) 1.63-1.60 (m, 4H), 1.32-1.28 (m, 48H), 0.89 (t, 6H, *J*=7.0 Hz).

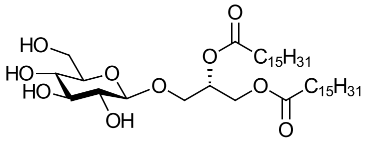

2

3-*O*-β-D-glucopyranosyl-1,2-di-*O*-hexadecanoyl-*sn*-glycerol (2, β-Glc-DAG)

To a solution of **S4** (4.6 mg, 0.004 mmol) in MeOH/toluene (1:1, 5 mL) was hydrogenated over Pd-black (23 mg) at 8 bar for 24 h using high pressure hydrogenation reactor. The solids were removed by filtration over a pad of Celite and the filtrate was concentrated to give **2** (2.8 mg, 91%). Analytical data were in accordance with the previously published data.[5]

^{1}H NMR (600 MHz, $CDCl_3$): δ [ppm] = 5.29-5.26 (m, 1H), 4.36-4.31 (m, 2H), 4.22 (dd, 1H, *J*=6.4 Hz, *J*=12.1 Hz), 3.92-3.90 (m, 2H), 3.81 (dd, 1H, *J*=5.1 Hz, *J*=11.9 Hz), 3.72 (dd, 1H, *J*=6 Hz, J=11.2 Hz), 3.57 (br. s, 2H), 3.40-3.31 (m, 2H), 2.34-2.30 (m, 4H), 1.62-1.58 (m, 4H), 1.29-1.25 (m, 48H), 0.88 (t, 6H, *J*=7.0 Hz).

References

1 Ye, X. S. & Wong, C. H. Anomeric Reactivity-Based One-Pot Oligosaccharide Synthesis: A Rapid Route to Oligosaccharide Libraries. *J. Org. Chem* **65**, 2410-2431, doi:DOI: 10.1021/jo991558w (2000).

2 Wong, C. H., Ye, X. S. & Zhang, Z. Assembly of Oligosaccharide Libraries with a Designed Building Block and an Efficient Orthogonal Protection-Deprotection Strategy. *J. Am. Chem. Soc.* **120**, 7137-7138, doi:DOI: 10.1021/ja9813616 (1998).

3 van Boeckel, C. A. A. & van Boom, J. H. Synthesis of phosphatidyl-α-glucosyl glycerol containing a dioleoyl phosphatidyl moiety. Application of the tetraisopropyldisiloxane-1,3-diyl (tips) protecting group in sugar chemistry. part III11For part I and II see references 11 and 39, respectively. *Tetrahedron* **41**, 4545-4555, doi:10.1016/S0040-4020(01)82349-8 (1985).

4 Isaad, A. L. C. *et al.* A hydrophobic disordered peptide spontaneously anchors a covalently bound RNA hairpin to giant lipidic vesicles. *Org. Biomol. Chem.* **12**, 6363-6373, doi:10.1039/C4OB00721B (2014).

5 Ohta, N. & Achiwa, K. Synthesis of biologically active galactosyl and glucosyl derivatives. *Chem. Pharm. Bull.* **39**, 1337-1339, doi:10.1248/cpb.39.1337 (1991).

NMR spectra of synthetic compounds

S3 ^{1}H NMR (600 MHz, $CDCl_3$)

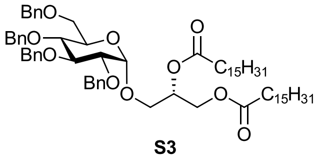

S3

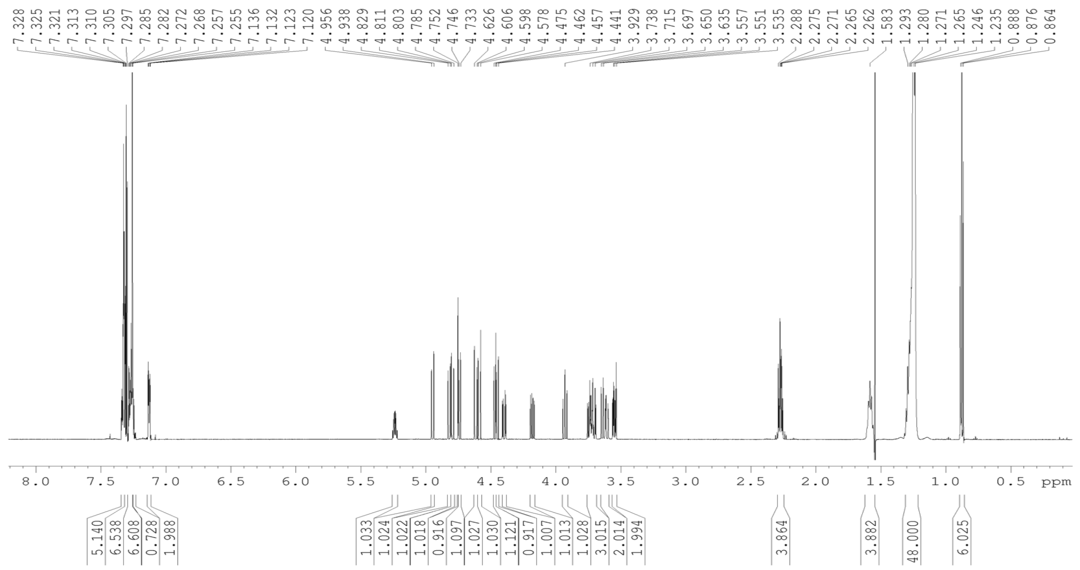

S3 ^{13}C NMR (151 MHz, $CDCl_3$)

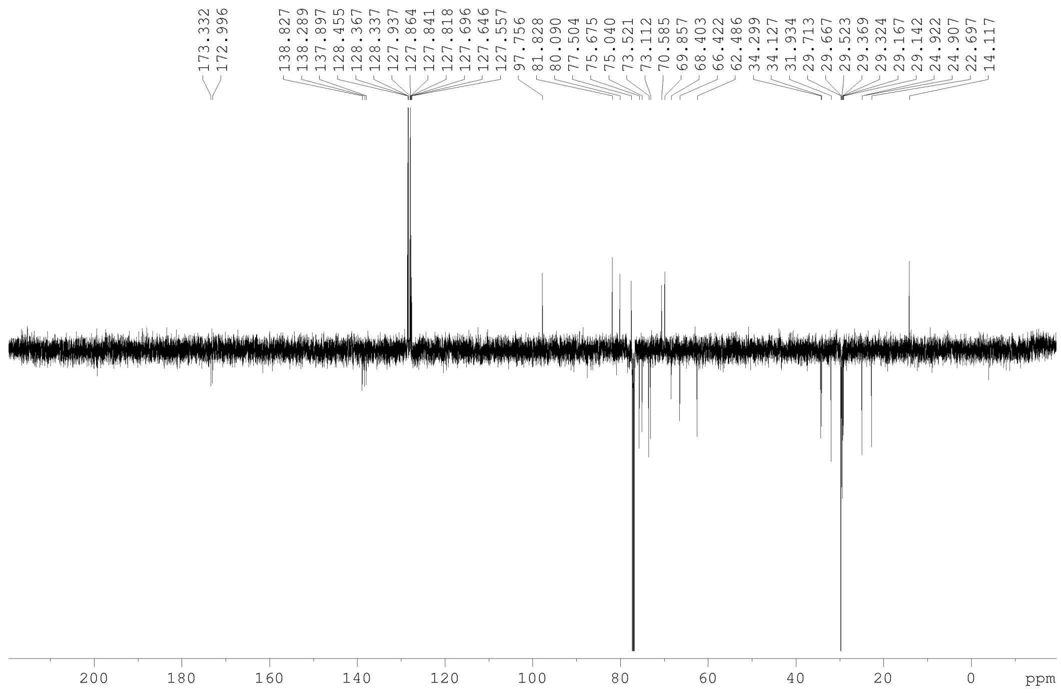

S3 HSQC NMR ($CDCl_3$)

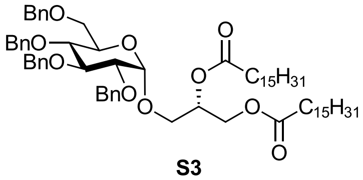

S3

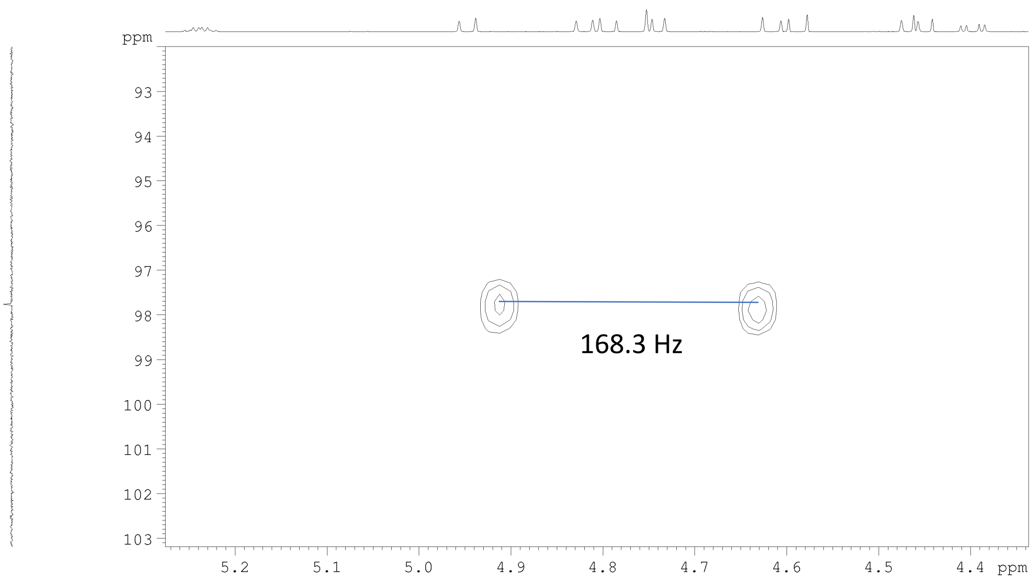

S4 ^{1}H NMR (600 MHz, $CDCl_3$)

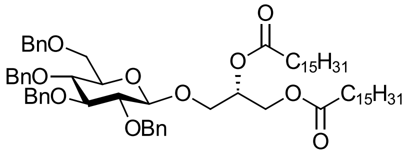

S4

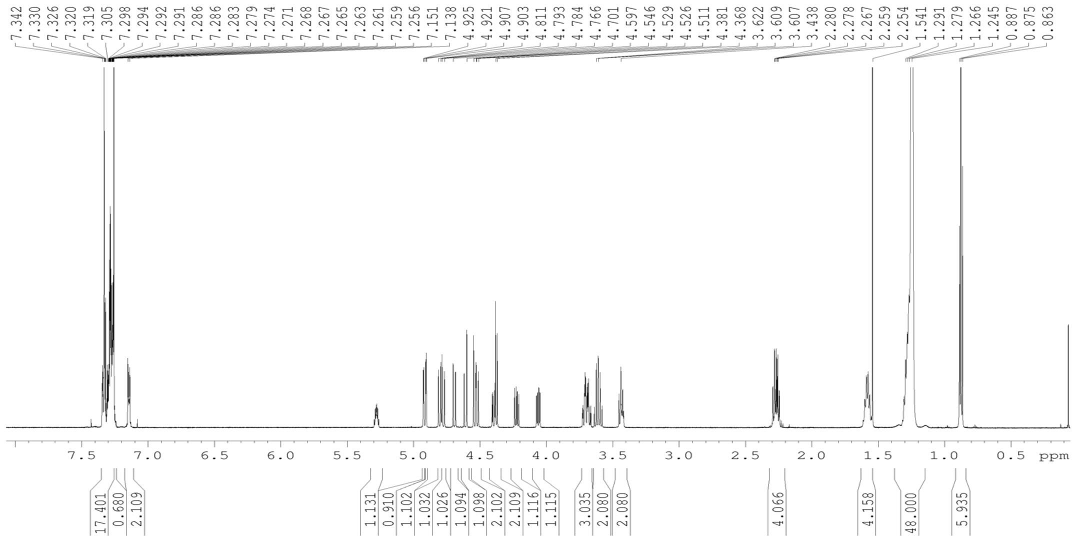

S4 ^{13}C NMR (151 MHz, $CDCl_3$)

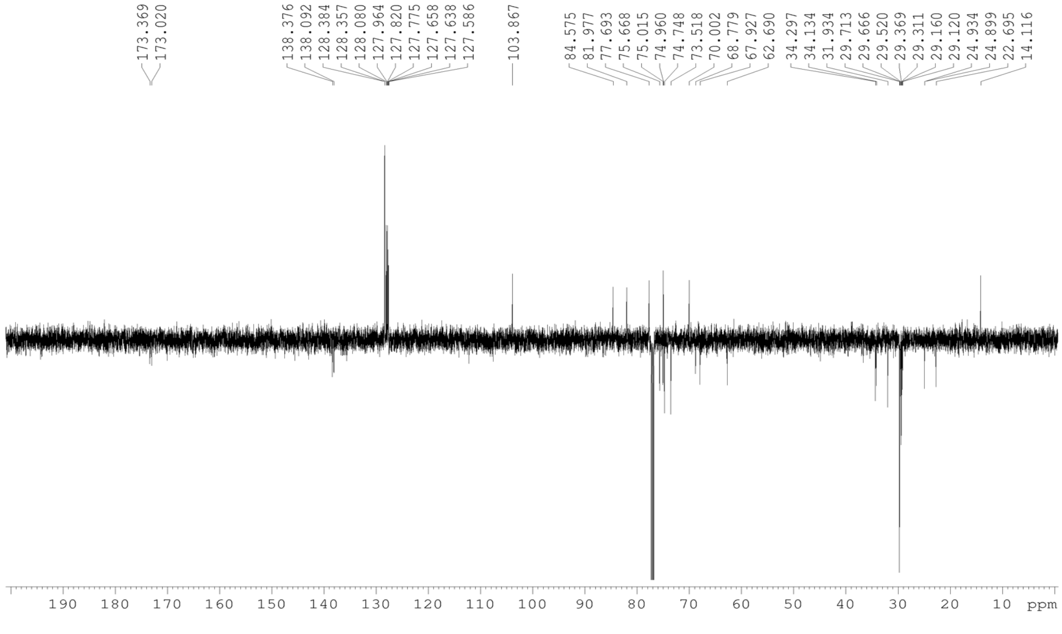

S4 HSQC-NMR ($CDCl_3$)

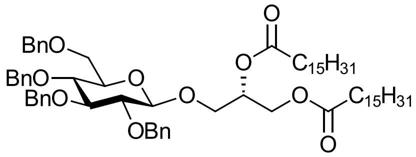

S4

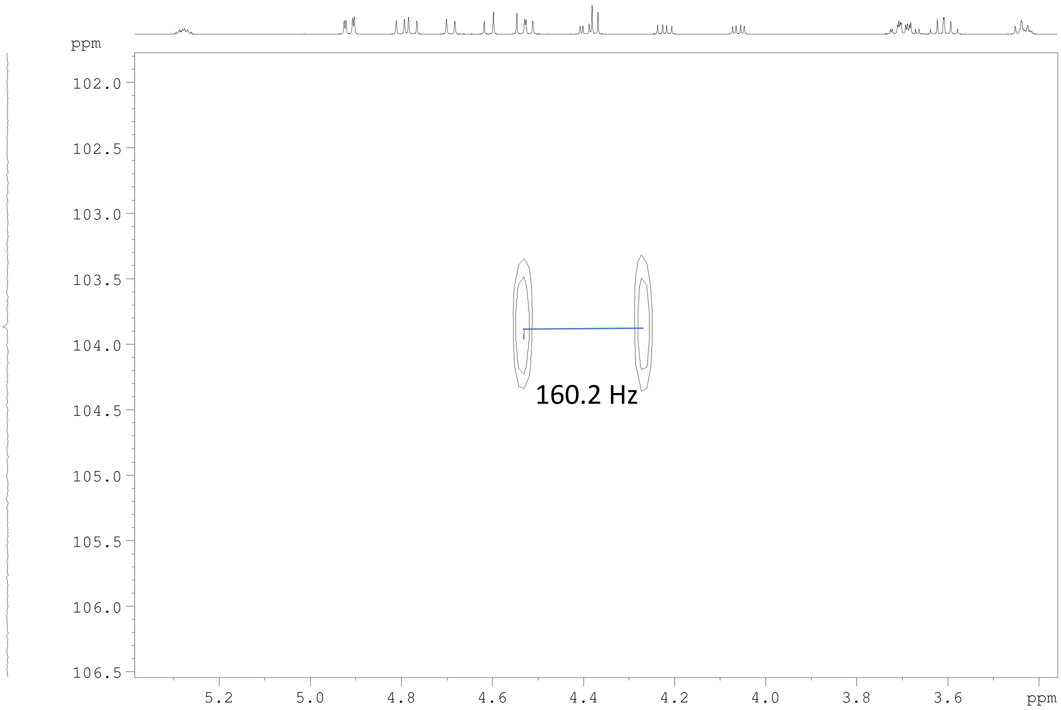

1 ^{1}H NMR (600 MHz, $CDCl_3$+CD_3OD)

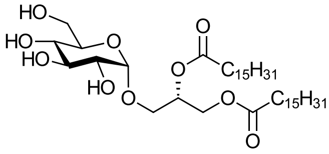

1

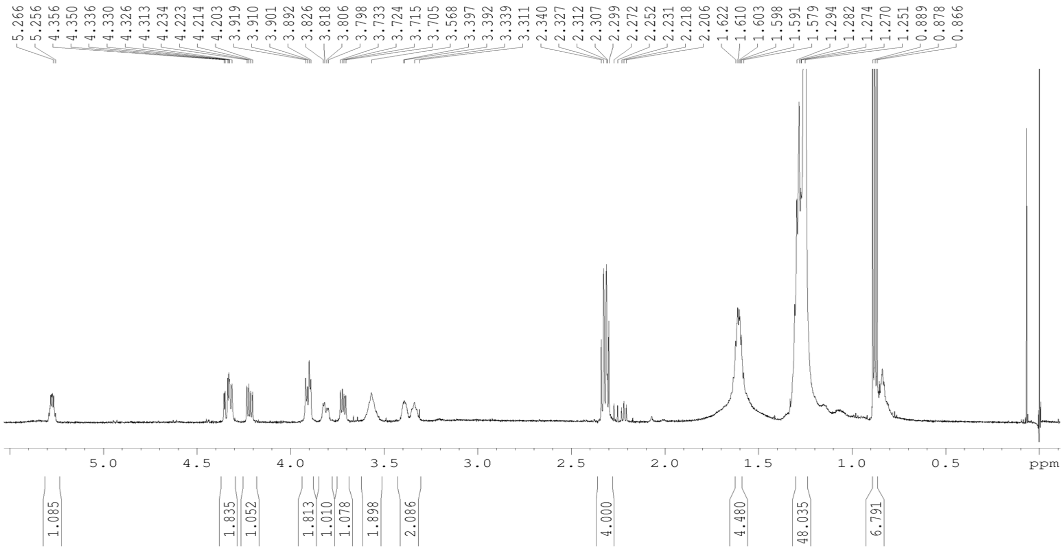

2 ^{1}H NMR (600 MHz, $CDCl_3$)

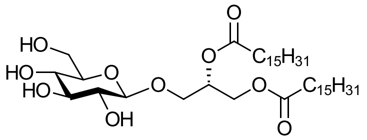

2

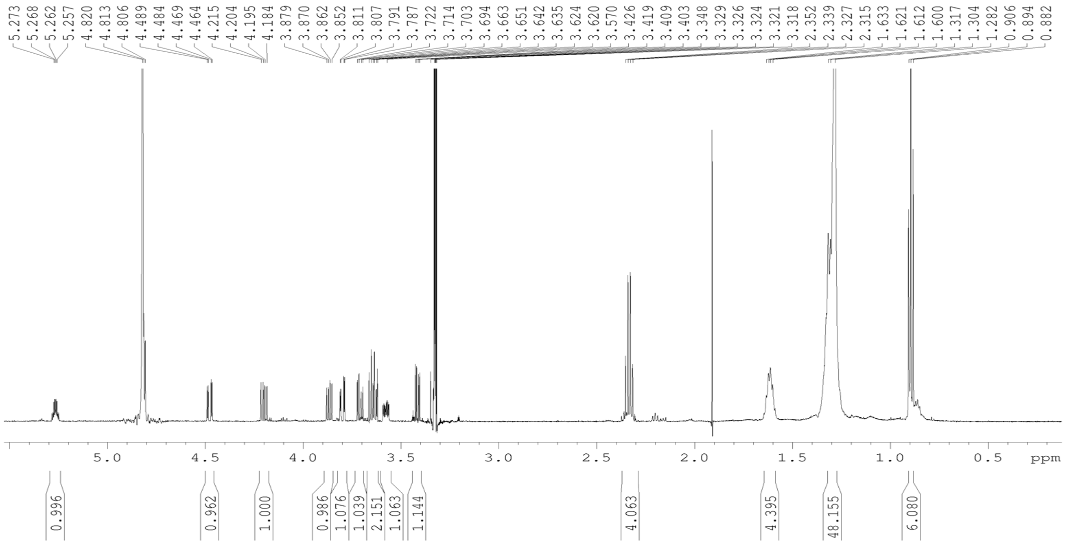

Mf1 sequence alignment with five selected proteins from a BLASTP search.

Consensus

1. BAH69265.1 cholinephosphotransferase Mf1 Mycoplasmopsis fermentans
2. WP 349222499.1 LicD family protein Roseburia inulinivorans
3. WP 063626040.1 diacylglycerol cholinephosphotransferase Mf1 Mycoplasmopsis gallinarum
4. WP 027334422.1 diacylglycerol cholinephosphotransferase Mf1 Mycoplasma elephantis
5. MFR1766058.1 MAG: phosphorylcholine transferase LicD Lachnospira sp.
6. MBP3452833.1 MAG: LicD family protein Clostridia bacterium

```
    ------XXXXXXMXEXQXXXLEXLXEFXXICXXXNLXYXXXYGTLLGAXRHXGFIPWDDDIDXXIXXXXY
             10        20        30        40        50        60        70
1   --------MIKTMNEKQTKVYELLKEFIEICNKHNLDYCAMYGTALGAIRHNGFIPWDDDIDLLISIETY
2   ------MTELT-MREIQLVSLEILHTVATICEEQNFRYSLIYGTLIGAIRHHGYIPWDDDVDIMMPRRDY
3   MHSNSEIKLCNEIKTRQTKNFELLLEFDQICKANNLFYTLAYGTALGAIRDQNLIPWDCDIDVIIDLKTY
4   ------------MTNKQIKVLELLKEFIEIADKHNLEYFVFYGTLLGAKRHKGFIPWDDDIDLIIPKKTY
5   ------MKQIVEMREMQSAALEVLKEFARVCEEQNLRYFLVYGTLIGAVRHNGFIPWDDDIDVMMPRKDY
6   -----MEEKKLTLEEIHEELLCQLRDIVAICNRHGIEYNMMCGSLLGAVRHKGFIPWDDDIDLLITREEF

    XXLXEXYXXXXXXX---------XXXXXXXXXXXXXXXXDXXXXXXXX-----XFXDXFXXXXXXXXXXK
             80        90       100       110       120       130       140
1   KFLKENYRNLIVDW---------ETGNSFLMFPKFSKDKDS---QKDV-----TFLDLFIDIPTTKKRIK
2   DQFLEYMKIHREEYPDLRIFNLEECKEYPYMITRISDDRYIIKMENEKPYGMGVFIDIYPYDGLGTTEKE
3   EFLKKHYANRILDN---------QYSHHCYQFPRFIFANDVDEKNEKP-----IFIDMFILIPTTIKKVK
4   DFLLENYSHKIKHN---------YNSNNFLLVSKFSNDNDT---NIDA-----VFLDLFVVVKTNKKNIK
5   DKLLEYYKAHDSKDSVFKLFNMDTCADYPYMISRLCDTRYYIDTDNEKDCGMGVFVDIYPYDGLGTDYAG
6   EKFRKIYPQECDKR---------FELTYLNTWTPRVMNRDP---EKAA-----AFTDFFILDSVPAEGWK

    XXXXXLKXXXXXXXXXXXRXXXXXXXXXKXXXXKXXXFXXXXXXKXXXXXXXXXXXEXXXX---XXXXXX
            150       160       170       180       190       200       210
1   KYLS-LKNKLGYLNAYSQRKFFDIQWGMK--IAKVLLFWTRMFKKPNYQKALETLYEPNSN------IHH
2   AVTYGMKGDRLSSLCYQSTREYFAIETTTSLFRKIIKYPVFIGSKLIGKDYFQKKLNQLVG---LKEYED
3   KYINSFKNKADATKGYFNRNIFFIQPKLMKFVQKLLKFFLFKNKTLTIDSSINQLLESNWNKNDPDSLYV
4   KFLS-LNTKIRYLRTFTYRRTFKCQWGIK--FLKCFLWWTLLWKKILFNDAWEQLYDENGN------LEM
5   AVKYALKGDRLSSACFQSTRLRYTTKNTKSIIRNIIKFPVFIISKIMGKEHYRRKLENLAG---RYDYES
6   RKWWFLKLH--ILQGMMKRNVDYSRFSLKN---KIMLFGTHLLGLPFSTEWKAKMYEKVSMAN-----PA

    XXXXXXXXXXXXXXXXIXXXXXFXDXIXVXFEDXXXXXPKXXXXXLXXXYGXXXXXPXXXXXXEXXGXXX
            220       230       240       250       260       270       280
1   VINWPF---KKATKINIYENINFFDTIDVKFEDIKIRVPKNYETIFLQNYGPNWRIPKKYKMSEHLGMYD
2   SNYIGCVVWLSGGKKEIFLRKWFDETIMVPFEKYQFRVPKDYDEVLRHTYGDYMQLPPE---KDRIGHHY
3   LSIWPSNFKKIKNQHLLKNQYVLNDLTTVSINNHQFSFFKDKEVIMEQWYGPNWKIPIQEKNWYFYGYYE
4   IIFWPS---KKDVYCNVYNEIDFSDYEFEKFEDIKVKIPKNWENALVKNYGVNWQIPIKFKLSEHLGMYD
5   SEYAACVVWLTGGEKDIFQREWFDDYIMMDFEDAKFRIPKEYDKVLRHTFGDYMQLPPK---SEQTPHHF
6   SNDLCMSNGAFELMMHIWHPDQFKEKITVKFEDVDVLIPAKHHEVLTTLFGPDYMTPPP--VEERVAKHL

    XXXXXXX--------
            290
1   MKVFCKKERAKKNDK
2   FKAYRKDE-------
3   KGKKKAD--------
4   MNVFTTK-RKK----
5   YKVYKK---------
6   DL-------------
```
